# Mapping of the Acoustic Environment at an Urban Park in the City Area of Milan, Italy, Using Very Low-Cost Sensors

**DOI:** 10.3390/s22093528

**Published:** 2022-05-06

**Authors:** Roberto Benocci, Andrea Potenza, Alessandro Bisceglie, Hector Eduardo Roman, Giovanni Zambon

**Affiliations:** 1 Department of Earth and Environmental Sciences (DISAT), University of Milano-Bicocca, Piazza della Scienza 1, 20126 Milano, Italy; a.potenza@campus.unimib.it (A.P.); alessandro.bisceglie@unimib.it (A.B.); giovanni.zambon@unimib.it (G.Z.); 2 Department of Physics, University of Milano-Bicocca, Piazza della Scienza 3, 20126 Milano, Italy; eduardo.roman@mib.infn.it

**Keywords:** eco-acoustics, cluster analysis, urban parks, environmental acoustic mapping, very low-cost acoustic sensors

## Abstract

The-growing influence of urbanisation on green areas can greatly benefit from passive acoustic monitoring (PAM) across spatiotemporal continua to provide biodiversity estimation and useful information for conservation planning and development decisions. The capability of eco-acoustic indices to capture different sound features has been harnessed to identify areas within the Parco Nord of Milan, Italy, characterised by different degrees of anthropic disturbance and biophonic activity. For this purpose, we used a network of very low-cost sensors distributed over an area of approximately 20 hectares to highlight areas with different acoustic properties. The audio files analysed in this study were recorded at 16 sites on four sessions during the period 25–29 May (2015), from 06:30 a.m. to 10:00 a.m. Seven eco-acoustic indices, namely Acoustic Complexity Index (ACI), Acoustic Diversity Index (ADI), Acoustic Evenness Index (AEI), Bio-Acoustic Index (BI), Acoustic Entropy Index (H), Normalized Difference Soundscape Index (NSDI), and Dynamic Spectral Centroid (DSC) were computed at 1 s integration time and the resulting time series were described by seven statistical descriptors. A dimensionality reduction of the indices carrying similar sound information was obtained by performing principal component analysis (PCA). Over the retained dimensions, describing a large (∼80%) variance of the original variables, a cluster analysis allowed discriminating among sites characterized by different combination of eco-acoustic indices (dimensions). The results show that the obtained groups are well correlated with the results of an aural survey aimed at determining the sound components at the sixteen sites (biophonies, technophonies, and geophonies). This outcome highlights the capability of this analysis of discriminating sites with different environmental sounds, thus allowing to create a map of the acoustic environment over an extended area.

## 1. Introduction

Changes in the natural landscape are generally prompted by induced modifications of encroaching urbanisation. Such modifications may impact the environment at different levels: from the growth of urban heat islands [1], to greenhouse gas emissions [2] and noise pollution [3,4]. All these effects may have an impact on biodiversity [5] with the resulting necessity for different species to adapt or undergo depletion [6]. Urban parks are particularly impacted by biodiversity imbalance because of the lack of gradients between natural and built environments which are often classified in a dichotomous fashion [7].

The study of environmental sounds has grown in importance as non-invasive techniques for ecological monitoring and have been recognised to convey important information on ecological status, such as species presence, environmental conditions and habitat quality [8]. This emerging discipline, known as eco-acoustics [9], investigates the soundscape that can be defined as the collection of sounds of different natures (geophonies, bio-phonies, anthrophonies or techno-phonies) that emanate from landscape [10,11,12].

Climate change and habitat degradation due to chemical pollution and technophonic noise sources may affect natural animal populations. Anthropogenic noises are always regarded as a pollutant for the environment as animal communication could be hampered, thus potentially causing changes in their distribution. For this reason, mapping biophonic activities on medium–large areas may help monitor the consequences of the spread of urbanisation.

The diffusion of the eco-acoustic approach among researchers has been favoured by the possibility of analysing acoustic recordings and converting them from a temporal domain to a frequency domain (e.g., after Fourier transforms). Several indices implemented on a matrix of frequency intensities have been published in recent years (for a review, see [13]). They are able to explain observed changes in habitat status [14], predicting species richness across a wide range of terrestrial [15,16] and aquatic habitats [17,18].

The need for cost-effective soundscape assessment tools is not limited to traffic noise mapping in urban areas, but it is a requisite across all fields of eco-acoustics and bioacoustics. The increasing memory storage and decreasing cost of hardware makes acoustic surveys comparable to satellite monitoring in terms of scalability in space and time, but has the potential benefit of providing a real picture of local population dynamics.

Although sounds are environmental components quite known at the level of individual species, they have not yet been implemented to monitor ecological dynamics on a large scale. In particular, sounds may play a primary role to describe the composition, diversity and dynamics of communities [19], and to assess the distance of ecosystems from healthy conditions [20]. Thus, acoustic surveys are becoming attractive for large-scale ecological monitoring, especially in remote locations because they are non-invasive, obviate the need for expert aural identification of individual recordings, are potentially sensitive to multiple taxa, and scale cost-effectively [13,19].

Recently, cost-effective soundscape assessment tools have successfully been applied to traffic noise mapping in urban areas, examples of which can be found for the cities of Madrid [21], Rome [22], Paris [23], and Rotterdam [24]. In particular, in ref. [25], the authors developed the dynamic mapping of traffic noise over a large area based on a limited number of monitoring stations. This result could be achieved by statistically grouping roads with similar traffic noise behaviour. The idea of extending this concept to natural environments is now becoming a strong requisite across all fields of eco-acoustics and bio-acoustics. In this regard, managing the influence and impact of urbanisation on green areas by monitoring across spatiotemporal continua could provide biodiversity estimation and useful information for conservation planning and development decisions. The capability of eco-acoustic indices to capture different sound features has been harnessed to identify areas within the Parco Nord of Milan characterised by different degrees of anthropic disturbances and biophonic activity. The area of Parco Nord of Milan has been the object of previous studies. More specifically, in ref. [26], the analysis of a 60 h recording allowed to distinguish among different biophonic activities during the day by applying an unsupervised statistical analysis. A further study based on the analysis of the auto-correlation between the time series of the computed eco-acoustic indices at two sites in the same area of Parco Nord allowed extracting indications on the environmental quality of the park [27]. Here, we are putting together the concept of spatial mapping developed in [25] with the information on the sound characteristics carried by different eco-acoustic indices as developed in [28] to obtain a spatial map of different sound blends over an area of approximately 20 hectares (not previously studied). To make this task cost-effective, we employed a network of very low-cost sensors distributed on a regular grid to highlight areas with different acoustic properties. The analysis described in this paper represents a contribution to establish a methodological approach in developing maps of environmental sound and eventually of environmental sound “quality”.

The outline of this paper is as follows: in Section 2, we describe the materials used—namely the area of study and the instrumentation, the statistical procedure applied on the eco-acoustic indices to derive a map of different environmental sounds, and the sound-truthing methodology to validate the analysis. In Section 3, we illustrate the results of the statistical analysis applied to the set of recordings over the study area. Section 4, we compare the obtained results with the labelling performed by an expert operator by hearing the recordings as a validation method. Finally, Section 5 summarises our general remarks and the possible developments we envisage.

## 2. Materials and Methods

In this section, we describe the Parco Nord area under study, the instrumentation used, and the data acquisition scheme. We also include the analysis methodology used for the analysis of the recorded signals based on the calculation of the eco-acoustic indices and the statistical process of obtaining a spatial map of different sound activities in the area. In the last part, we describe the adopted sound-truthing with all the categories of sounds considered for the validation of the results.

### 2.1. Area of the Study

The Parco Nord of Milan expands over an area of approximately 790 hectares all the way up to its northern border and is encircled by a highly urbanised area. About 45% of the surface is devoted to green spaces and vegetation: wooded areas (over 100 ha), meadows, shrubs, hedges, and ponds. The remaining is dedicated to agricultural activities and infrastructures. More than 100 species of trees, shrubs and ornamental plants grow in the area.

The chosen area of study consists of a tree-covered parcel of approximately 20 hectares. It is an area dating back to 1983, one of the first reforestations implemented to restore the area intended to become a park. This parcel of wood has been thinned out over time to favour the regeneration of the wood itself. Nowadays, it has a semi-natural structure, with herbaceous layers of nemoral flora, shrub layers and the presence of dead woods. There is a small body of water (an artificial lake of approximately 300 m${}^{2}$) which is approximately 250 m from the edge of the bush. Visitors use this area mainly for walking or playing sports, as it is crossed by numerous paths, and the neighbouring areas with no trees for recreational activities.

A more specific characterisation of the area of study, in terms of vegetation coverage, was performed through the analysis of satellite images collected as part of the Landsat-8 NASA mission [29]. NASA has developed different Landsat missions with polar-orbiting satellites placed in the same sun-synchronous orbit at an height of 705 km and specifically designed to provide data for agribusiness, global change researchers, academia, state and local governments, commercial users, national security agencies, the international community, decision makers, and the public. With its 11 spectral bands, 185 km swath width, high revisit frequency and spatial resolution of 15 m, 30 m, and 100 m according to the chosen frequency band, Landsat supports a wide range of land studies and programs.

The image downloaded refers to the period of 10–17 May 2015. A map of the Normalised Difference Vegetation Index (NDVI) [30] was calculated for each downloaded image in the GIS environment [31], for the preliminary characterisation of the area. NDVI is one of the main indicators, obtained from satellite images, of the presence of vegetation on the Earth’s surface and its evolution over time. The index evaluates the presence of photosynthetic activity, as it relates the spectrum of red, in which there is absorption by chlorophyll, and that of the near infrared, in which the leaves reflect light to avoid overheating. Index values are typically between −1 and +1. The presence of vegetation yields values greater than 0.2. The NDVI index is calculated as follows:

(1) $$\mathrm{NDVI}=\frac{\mathrm{NIR}-R}{\mathrm{NIR}+R}$$

where NIR and R are the spectral reflectance measured in the near infrared and red wavebands, respectively. NDVI values vary between −1 and 1; values <0 are associated with presence of water, values <0.4 with soil and >0.4 with vegetation. The interpretation of this index strongly depends on the area under examination, but is generally interpreted as reported in [32]. This index will be used to identify potential correlation between vegetation coverage and biophonic activities in an area of study of the parco Nord of Milan.

As for the acoustic characterisation of the area, many sources which may bring to a partial masking of the singing activity of the avian community are present. We can identify them as: noise generated by park users, vehicular traffic, road works, and occasional overflights of the nearby Bresso airport. The birds detected in recent surveys in all the park belong to 39 different species.

### 2.2. Instrumentation

As highlighted in the Introduction, the main purpose of this work was to investigate the sound environment within a homogeneous plot of land (a wooded parcel) with the aim of quantifying the spatial variations at an increasing distance from technophonic sound sources. Creating maps of environmental sounds over a large area may require important financial commitment that could benefit from the availability on the market of very low-cost recorders (VLCRs). We used SMT security digital audio recorders with a 48 kHz sampling rate and a power bank lifetime of two weeks.

#### Characterisation of VLCRs

One of the disadvantages of using very low-cost recorders is the possible different microphone sensitivities they may have, causing a different response to a sound exposure. For this reason, an initial set of 46 VLCRs (numbered R01–R46) was preliminarily tested in the laboratory to select sensors with similar behaviour. In fact, the use of very low-cost sensors occasionally implicates the presence of anomalies in the responses, both in the range of frequencies and in signal intensity. The mounting position of the sensor in the sound field can also lead to response differences. According to a testing procedure implemented ad hoc, all recorders were individually mounted on a stand and exposed to white noise generated by a calibrated flat loudspeaker placed at a fixed distance. All responses were recorded with the same directivity (normal angle of incidence between the source and body of instrument). The response of each VLCR was evaluated in terms of:

- Computation of the ACI index for white noise source.
- Behaviour of each VLCR at different frequencies.

We opted for this procedure because the ACI index provides an overall evidence of any anomalies in the frequency response. The process of the ACI index calculation (described in Section 2.4) computes the relative variation of recorded amplitudes of adjacent temporal steps in each frequency bin, as determined by the FFT analysis.

**Figure 1:**
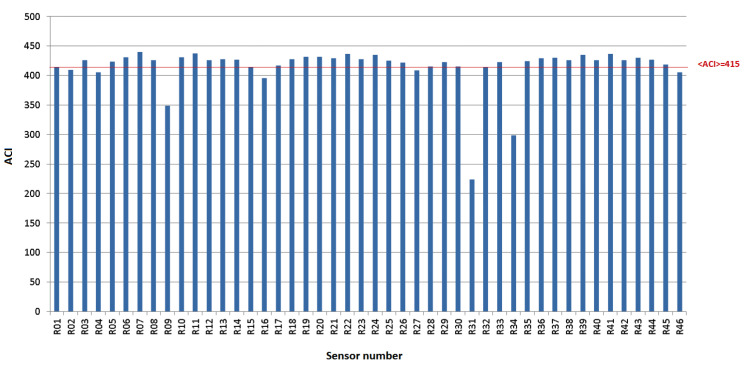
Response of all 46 sensors in terms of computed ACI. The red line represents the average value, <ACI>≈415.

Figure 1 reports the ACI index values, corresponding to the white noise recordings for all 46 sensors. Anomalies were identified when the recorder response strongly departs from the average value, <ACI>≈415. Based on these results, 22 VLCRs were selected. In particular, we considered variations within 3% from <ACI>.

**Figure 2:**
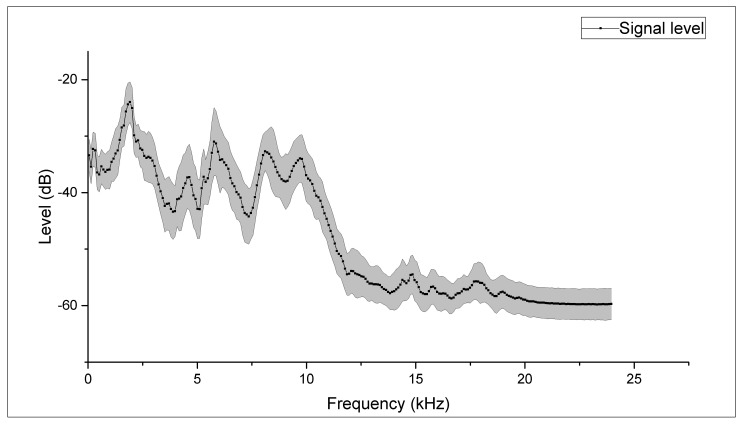
Frequency response of the 22 selected sensors: average spectrum and standard deviation (grey band). The frequency range is 0–24 kHz.

Figure 2 shows the average spectrum (computed using a 512-point FFT analysis) obtained from the 22 recorders using white noise as a sound source. It is worth noting that the frequency response is not flat over the entire frequency range with a marked drop in sensitivity in the region beyond 10 kHz.

**Figure 3:**
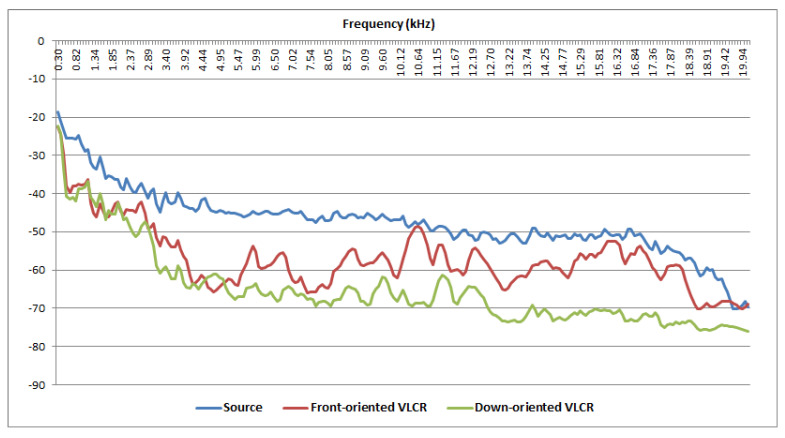
Frequency response of a single VLCR exposed at different orientations with respect to the incident sound source (blue line) generated with the Audacity software. The downward oriented response (green line) yields a better correlated response with the source signal due to the mounting position of the microphone embedded in the VLCR.

A further analysis was carried out to verify the response dependency on the incident orientation of the sound source. Two recorders were randomly chosen from the set of 22 VLCRs previously selected and they were exposed to a sound signal generated by Audacity, the first frontally oriented and the second downward oriented. Figure 3 shows that the downward oriented device (green line) yields a better correlated response (0.79 against 0.66 for the front-oriented VLCR) with the source signal (blue line) due to the mounting position of the microphone embedded in the VLCR.

### 2.3. Measurement Scheme

**Figure 4:**
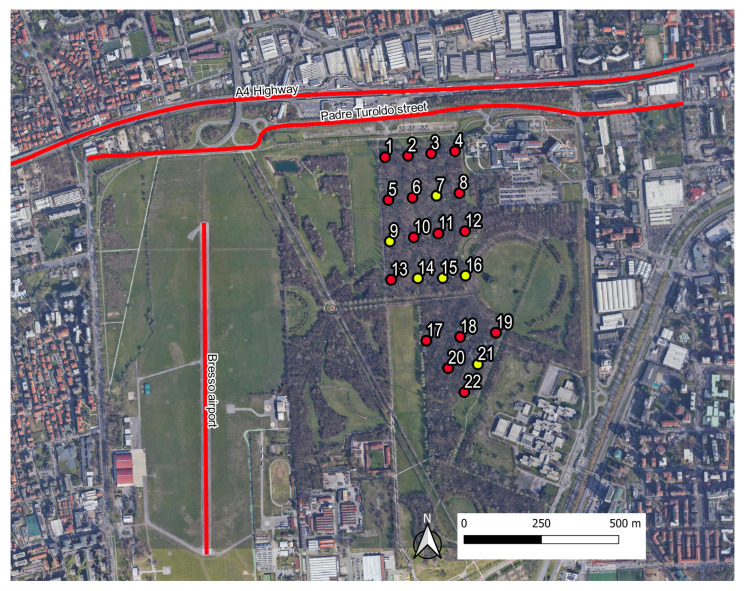
Area of study at the Parco Nord of Milan with indication of the measuring sites. Red spots indicate the active recording sites, yellow spots indicate sites where the recordings failed. Furthermore, the following are highlighted: the A4 highway, Padre Turoldo Street and the Bresso airport runaway.

The 22 recorders were initially positioned on a regular grid (see Figure 4) covering an area of approximately 75 × 135 m${}^{2}$ plus another grid with an area of 100 × 100 m${}^{2}$ for the southern part of the parcel. The recordings were scheduled for the period of greatest singing activity of the avifauna and repeated over four days, namely during the period of 25–28 May 2015, from 06:30 a.m. to 10:00 a.m., corresponding to 3.5 h for each site and for each recording session. Unfortunately, six recorders did not work properly (see yellow spots in Figure 4) and thus, the audio files analysed in this study were recorded at only 16 sites.

**Figure 5:**
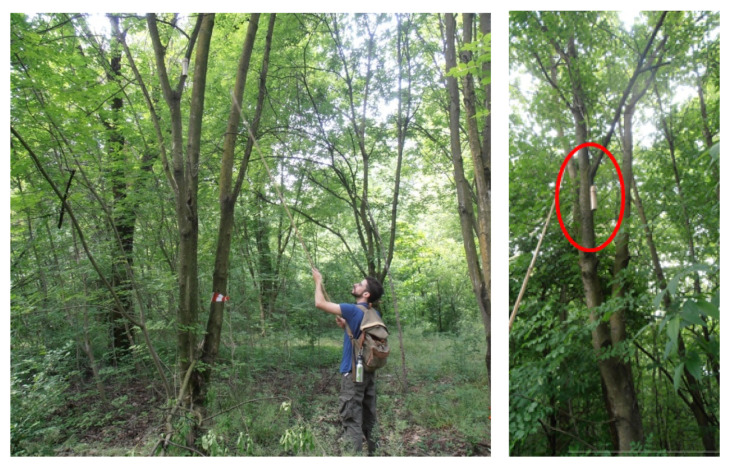
(**Left panel**) Deployment of very low-cost sensors in the Parco Nord bush. (**Right panel**) Blow-up of the recorder position highlighted by the red circle.

Figure 5 shows the phase of deployment of a very low-cost recorder inside the park (left panel) with a blow-up shown in the red circle (right panel).

### 2.4. Indices Computation

The “R” software (version 3.5.1 [33]) was used for the analysis of the file recordings and the calculation of eco-acoustic indices. Specifically, the fast Fourier transform (FFT) was computed by the function *spectro* available in the R package “seewave” [34] in the frequency interval (0.1–24) kHz based on 1024 data points which corresponds to a frequency resolution of FR = 46.875 Hz, and therefore, a time resolution TR = 1/FR = 0.0213 s. The eco-acoustic indices were computed using the R package “soundecology” [35]. A dedicated script running in the “R” environment was written to calculate the Dynamic Spectral Centroid (DSC) index.

In general, eco-acoustic indices were grouped into categories aiming to quantify the sound amplitude, its level of complexity, and weight the importance of geophonies, biophonies, and technophonies (soundscape). In this work, we focused on the following set of eco-acoustic indices:

- Acoustic Entropy Index (H), which highlights the evenness of a signal’s amplitude over time and across the available range of frequencies [19];
- Acoustic Complexity Index (ACI), which determines the modulation in intensity of a signal over changing frequencies [36];
- Normalised Difference Soundscape Index (NDSI), which accounts for the anthropogenic disturbance by computing the ratio between technophonies and biological acoustic signals [37];
- The Bio-Acoustic Index (BI), which is calculated as the area under the mean frequency spectrum above a threshold characteristic of the biophonic activity [16];
- Dynamic Spectral Centroid (DSC), which indicates the centre of mass of the spectrum [38].
- Acoustic Diversity Index (ADI), which provides a measure of the local biodiversity at the community level without any species identification [38].
- Acoustic Evenness Index (AEI), which provides reverse information of ADI with high values identifying recordings with dominance of a narrow frequency band [38].

For each site, the eco-acoustic indices were computed at 1 s integration time. The information in each time series was extracted using the most representative statistical metrics. For this purpose, seven descriptors were considered: the mean value, median, mode, standard deviation (SD), interquartile range (IQR), skewness, and kurtosis.

### 2.5. Statistical Analysis

In this subsection, we will present methodologies used for the analysis of the recorded signals.

#### 2.5.1. Principal Component Analysis

Principal component analysis (PCA) is a multivariate technique that analyses a dataset in which observations are described by several inter-correlated quantitative dependent variables, thus acting as a dimensionality-reduction method to reduce the number of variables or the dimensionality of large datasets [39]. The principal components are linear combinations of the original variables and are orthogonal to each other, meaning that correlated variables are merged together with different weights into new variables generally referred to as dimensions. Thus, components with low variance are less relevant and can be discarded. The general approach for selecting the optimal number of components is based on the cumulative percentage variance, which must be larger than 80% and the eigenvalue with a cut-off set at 1 (see [40]). Hence, in our case, after the calculation of the eco-acoustic indices, we end up, for each site, with 49 variables, i.e., 7 indices × 7 statistical descriptors. In order to reduce the number of variables, we first performed a principal component analysis (PCA) on the input dissimilarity (distances) matrix, which is of order 16 × 49, corresponding to the 16 observations (recording sites) times the 49 variables representing the computed eco-acoustic indices.

#### 2.5.2. Cluster Analysis

Cluster analysis is a set of techniques aiming to find patterns in a set of objects in such a way that objects in the same group or cluster are more “similar” to each other than to those in other clusters. It is used as an approach to summarise the main characteristics of a dataset and is a common technique for statistical data analysis such as pattern recognition, image analysis, information retrieval, bioinformatics, data compression, computer graphics, and machine learning [41].

Hence, searching for spatial patterns of different bio/anthropic activities in the area of study has been addressed by applying an unsupervised clustering analysis to group together the information carried by the selected principal components (PCs). Here, we employed the following clustering algorithms: ‘hierarchical’ agglomeration using Ward algorithm [42]; k-Means Algorithm [43]; Partitioning Around Medoids (PAM) [44]; Divisive Analysis Clustering (DIANA) [44]; Self-Organising Tree Algorithm (SOTA) [45]; Clustering Large Applications (CLARA) [44]; Agglomerative Nesting (Hierarchical Clustering) AGNES [44] and the corresponding results were compared. We searched for a range of clustering solutions between ten and two, the latter corresponding to minimal discrimination between the datasets. The metric used to evaluate the distance among observations is the Euclidean distance. The clustering computation was performed in “R” environment and the package “clValid” [46,47] was used as a guide for choosing the proper clustering algorithm [48,49,50,51,52,53].

### 2.6. Aural Survey

In this section, we describe the scheme adopted for the aural analysis of audio files in order to quantify distinct sound features. A single expert carefully listened to each recording according to the following scheme: one-minute listening to every two minutes of continuous recording, for a total of 70 min-listening per site. In particular, this activity was focused on determining biophonic activities (mainly avian vocalisation and other animals), technophonic sources (mainly noise from the highway, a construction site and airplanes taking off from nearby Bresso airport), human voices and steps and rain and wind sounds, as reported in Table 1.

Each recording was labelled according to the categories illustrated in Table 2. In particular, the label “perceived singing activity” is expressed in terms of percentage of singing activity in each one-minute recording.

## 3. Results

**Figure 6:**
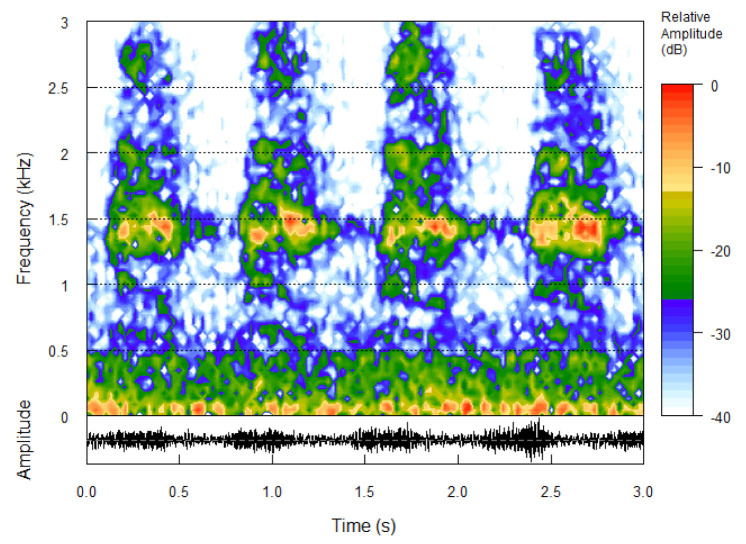
Carrion crow’s call spectrogram: the maximum relative amplitude is centred at approximately 1.5 kHz, extending up to 3 kHz. The frequency band between ≈0 and 0.5 kHz is due to traffic noise. Time scale: 0–3 s. Frequency range: 0–3 kHz.

The results presented in this section refer to the audio files recorded on 25 May 2015, from 06:30 a.m. to 10:00 a.m. The choice of eco-acoustic indices and their settings for the analysis requested an initial tuning due to the specific environment under investigation. We started with the seven eco-acoustic indices described in Section 2.4 which have also been employed in previous works [26]. For NDSI, the threshold between human-generated and biological sounds is generally set at 2 kHz, whereas for BI, the area under the mean frequency spectrum describing the biophonic activity is limited within 2 kHz and 8 kHz. These settings have been successfully used in other contexts [27], but in this specific case they proved to be inappropriate. Indeed, two indices, namely DSC and NDSI, are generally considered to be highly correlated as they carry information on the frequency content of recordings. On the contrary, our analysis, consisting of finding groups of sites with similar environmental sounds, revealed an opposite trend. We found that specific bird species such as the carrion crow (*Corvus cornix*), the wood pigeon (*Columba palumbus*), and the cuckoo (*Cuculus canorus*) produced sounds in the interval considered to be restricted to technophonies. Actually, the carrion crow’s call spectrum, which is the most active, is centred at approximately (1.3–1.4) kHz, as illustrated in Figure 6.

**Figure 7:**
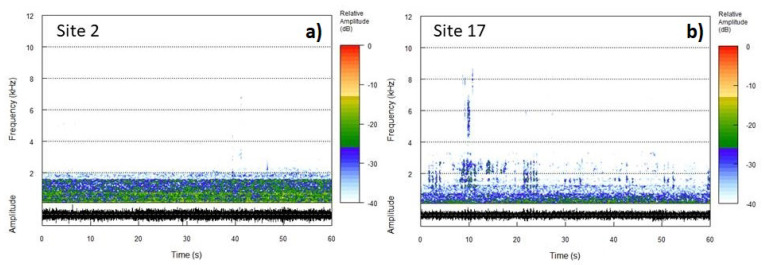
Spectrogram of two simultaneous recordings taken at Site 2 (panel (**a**)) and Site 17 ( panel (**b**)) showing the different contributions of traffic noise at the two sites.

However, adjusting the interval bounds for human-generated and biological sounds (threshold for NDSI to 1 kHz and interval for BI between 1 kHz and 8 kHz) was not sufficient to obtain satisfactory results in terms of groups of sites with similar sound features. Considering the spectrogram of two recordings taken simultaneously at Site 2 and Site 17 (see Figure 7), we can observe that the traffic noise contribution covers the spectrum differently. Indeed, for Site 2 (left side of Figure 7) which is closer to the highway traffic source, the higher frequency components of the traffic noise significantly exceed the threshold limit set for NDSI and BI at 1 kHz. This excess is less important for Site 17 (right side of Figure 7) because of the higher diffraction that higher frequencies undergo. Thus, this consideration poses serious limitations to the use of these indices in this specific context. For this reason, we limited our analysis to five eco-acoustic indices, namely ACI, ADI, AEI, H, and DSC. Therefore, we will refer to these indices hereinafter. Referring to the spectra shown in Figure 7, the calculated ACI, ADI, AEI, H, and DSC are reported in Table 3. Here, the effects of the presence of singing activity and less traffic noise background is reflected in the index values obtained for Site 17.

### 3.1. Results of the PCA

As reported above, PCA was performed in order to reduce the number of clustering variables and obtain an insight into the variable relevance. Here, we illustrate the results obtained for the statistical metrics describing the distribution of the indices.

**Figure 8:**
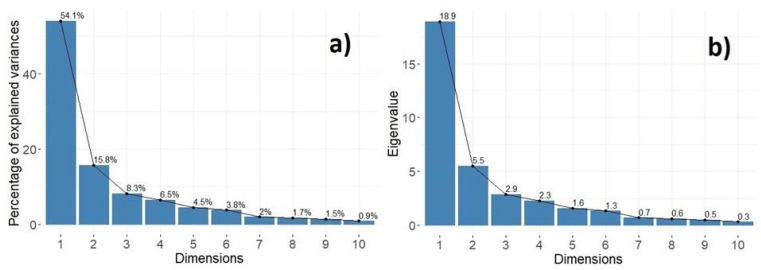
Properties of the first ten principal components: (**a**) percentage of explained variance vs. dimensions; (**b**) eigenvalues vs. dimensions. The cumulative contribution of explained variance, *V*, of the first three dimensions is 78.2%.

In keeping with the largest possible variance of the original variables, we selected the first 3 dimensions out of 35 original variables (5 indices × 7 statistical descriptors). Dimensions d=1 to d=3 have a cumulative percentage of explained variance, *V*, given by $V_{1}+V_{2}+V_{3}=\left(54.1+15.8+8.3\right)\%=$ 78.2% (Figure 8a). The corresponding eigenvalue for dimension three is $E_{3}=2.9$, (Figure 8b). The values V≥80% and $E_{c}\leq 1$ are commonly used as a cut-off in order to decide which principal components to retain. In our case, to reduce the total number of variables and not lose the possible interpretation meaning, we decided to lean on just the first criterion. Therefore, we hereinafter use the first three components (or eigenvectors, herein denoted as dimensions) of the PCA.

Table 4 reports the contributions of the variables in accounting for the variability of the first four principal components. It indicates that dimension 1, describing 54.1% of the data variability, is mostly associated with the statistical descriptors of ADI (29.8%), AEI (30.0%) and H (27.4%); DSC contributes to dimension 2 with 57.8%, and dimension 3 has a prevalence of ACI (41.5%). However, the first dimension carries most of the variance and its contribution is significantly represented by evenness indices.

### 3.2. Results of the Cluster Analysis

In this section, we present the results of the cluster analysis performed on the eco-acoustic indices computed as a 1 s time series whose distribution was studied through the most representative statistical metrics as described in Section 2.4. As discussed in the previous section, the first three PCA components were taken as input for the subsequent cluster analysis. A first approach to determine the most efficient clustering algorithm and the optimal number of clusters was to run the clValid cluster ranking algorithm. A solution with ten clusters was returned for different clustering algorithms. Thus, in keeping with excluding excessively fragmented results, as one can expect with a ten-cluster aggregation, we focused on DIANA and k-means algorithms with two, three, and four clusters. Observing the allocation of the measuring sites, the DIANA algorithm with two clusters provides a one-observation-cluster with the rest of observations allocated in the other one.

Actually, as a divisive hierarchical clustering algorithm, DIANA starts with one large cluster containing all *n* observations and is then divided until each cluster contains only a single observation. At each stage, the observation with the largest average dissimilarity is separated (top–down process). In our case, Site 18 is separated from the other sites. On the contrary, k-means operates the other way round, as an agglomerative algorithm (bottom–up process). As such, the two clusters are almost equally occupied (cluster 1 with seven observations and cluster 2 with eight observations). We also tested the solution at three and four clusters to possibly highlight more complex patterns in the acoustic data. In this case, both DIANA and k-means algorithms provide the same outcome with Site 18 to form a single cluster for both the two options (three and four clusters).

**Figure 9:**
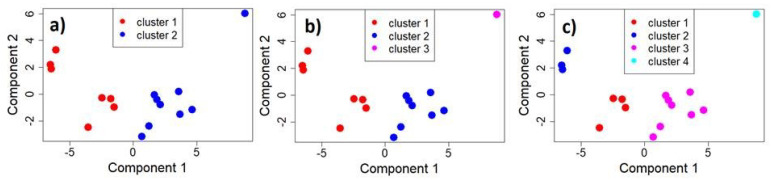
Multi-dimensional scaling (MDS) applied to the k-means clustered data to provide a visual representation of the pattern of proximities among data. (**a**): two clusters; (**b**): three clusters; and (**c**): four clusters.

**Figure 10:**
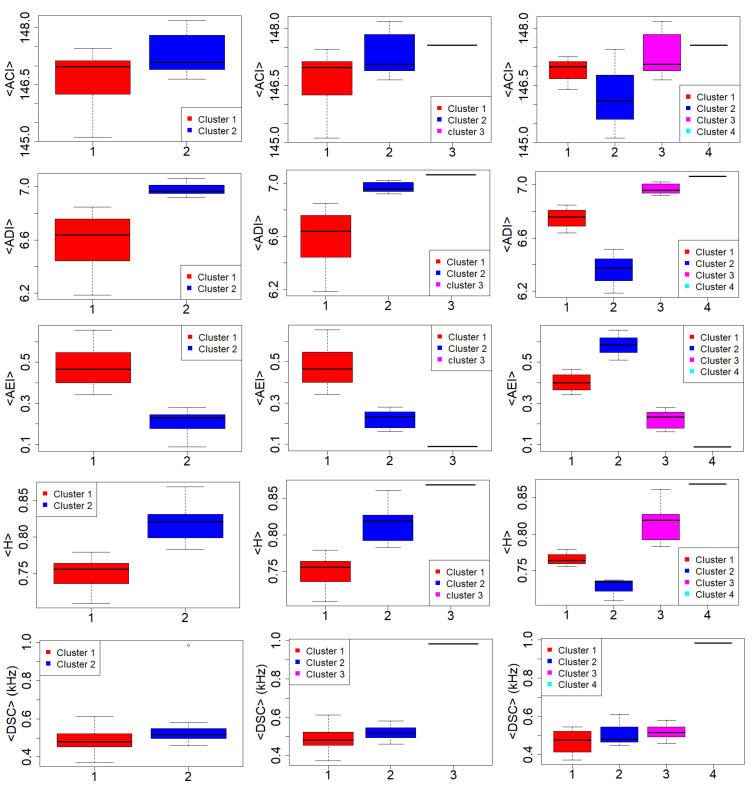
Boxplot of the indices’ mean value for the two (**left column**), three (**central column**), and four-cluster decomposition (**right column**). k-means is the clustering algorithm. From **top** to **bottom**: <ACI>, <ADI>, <AEI>, <H>; and <DSC>.

Figure 9 illustrates the multi-dimensional scaling (MDS) results applied to the k-means clustered data to provide a visual representation of the pattern of proximities among the data [54] for the three selected solutions. The distinction between clusters, marked by different colours, is rather satisfactory. Figure 10 illustrates the boxplots of the original mean eco-acoustic indices split into the different cluster: 2 (left column), 3 (central column), 4 (right column).

For a straightforward interpretation, we considered the distribution of mean values of each index into the obtained clusters. As can be clearly seen, the statistical analysis based on a reduced number of dimensions is able to quite efficiently separate the mean-index values based on the k-means algorithm. This result reflects the robustness of the clustering after the substantial dimension reduction.

In order to evaluate whether these differences are significant, we applied the Wilcoxon–Mann–Whitney test (WMW) [55] to the two-cluster solution and the Kruskal–Wallis test (KW) [55,56,57], to the three and four-cluster solution as not all the set of data satisfy the Shapiro–Wilk’s test on normality [58]. Both tests are non-parametric statistical methods used to enquire the hypothesis of whether two or more samples are drawn from the same distribution. Here, the null hypothesis, $H_{0}$, says that there is no significant difference between two (WMW) or more than two (KW) populations or variables. In contrast, the alternative hypothesis, $H_{1}$, states the opposite outcome. Table 5 reports the results of the WMW test applied to the two-cluster solution at the 95% significance level. Similar results were obtained for the KW test applied to the three and four-cluster solution. For all cases, the statistical descriptors associated with the distribution of mean values in the obtained clusters are statistically different with the exception of ACI and DSC indices. This means that the real difference among clusters is due to evenness descriptors.

**Figure 11:**
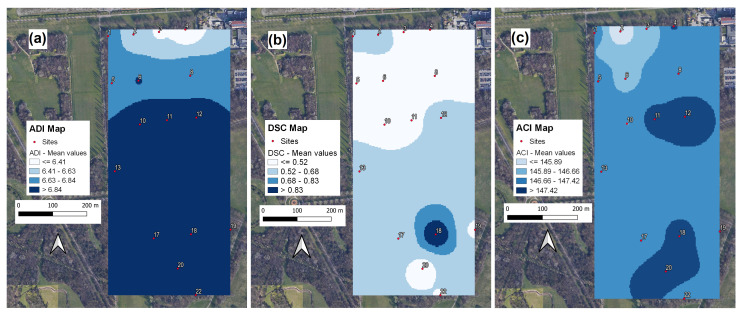
Spatial maps corresponding to: (**a**) ADI; (**b**) DSC; and (**c**) ACI. Colours refer to four equally spaced classes of values.

The spatial distribution of the different acoustic descriptors representative of Dimensions 1, 2 and 3 (see Table 4) is illustrated in Figure 11. In particular, <ADI> (see Figure 11a), which is representative of Dimension 1, highlights (together the correlated AEI and H indices) the road traffic noise impact. Dimension 2, represented by the DSC index and illustrated in Figure 11b, highlights Site 18, whereas the spatial distribution of ACI (representative of Dimension 3) is illustrated in Figure 11c. Here, we can see how higher frequency modulations, most likely due to singing activity, are found in the inner part of the park and far from the annoying traffic source.

### 3.3. Results on the NDVI Index

**Figure 12:**
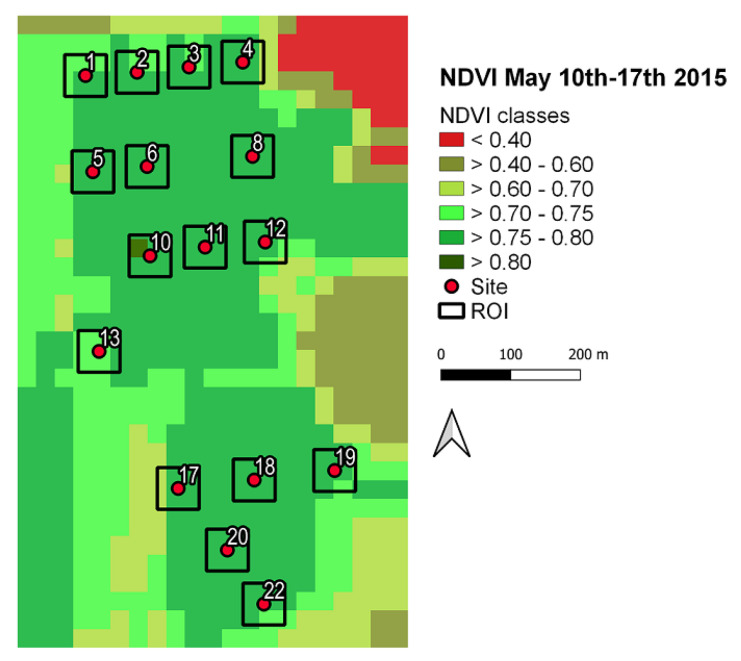
NDVI index computed for the period 17–19 May 2015. The considered ROIs are squares centred around each site with a 60 m side. Different colours refer to different vegetation coverage.

In this section, we present the results on the NDVI index computed for the period 17–19 May 2015. The NDVI index is usually employed to analyse remote sensing measurements and assess whether the observed area contains live green vegetation, as described in Section 2.1. For this purpose, a region of interest (ROI) corresponding to a square area of a 60 m side centred around each site (see Figure 12) was considered. The figure also illustrates the variability of the index according to different colours.

## 4. Discussion

In this section, we will combine the results of the statistical analysis performed on the eco-acoustic indices in order to determine a spatial map of environmental sound in the area of study.

**Figure 13:**
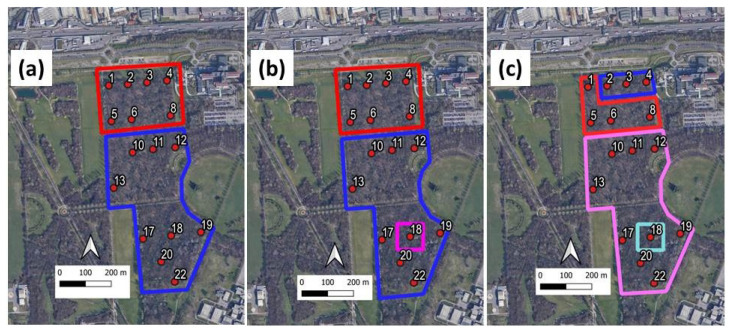
Areas corresponding to: (**a**) two, (**b**) three, and (**c**) four-cluster solutions. The colour contours are drawn as help for the eyes with the following correspondence: red = cluster 1; blue = cluster 2; pink = cluster 3; light blue = cluster 4. The reported sites represent the effective measuring sites.

In Figure 13, the areas corresponding to the two-, three-, and four-cluster solutions are highlighted in different colours. An important feature of the analysis is the continuity of the sites, meaning that the environmental sound is not fragmented but enclosed in specific areas. With two-cluster solutions, we observe a sharp distinction between the sites closer to the traffic noise source represented by the nearby highway and the other sites with a more central position. Observing the left column in Figure 10, we can see how cluster 1 presents lower indices values, the expression of less frequency modulation (ACI = 146.97) against cluster 2 (ACI = 147.9) (see Table 6) and a lower frequency bin occupation expressed by ADI index (opposite trend for AEI) with ADI = 6.64 against 6.96 for cluster 2. Indeed, ADI measures entropy in frequency space and higher ADI values indicate greater homogeneity across frequency bands while lower ADI values highlights the presence of an unbalanced frequency distribution in the spectrum.

Similarly, the acoustic entropy index, H, is related to frequency homogeneity over the spectrum and its variation over time. This means that high values of H are correlated with higher frequency occupation of the spectrum and time. In our specific case, cluster 1 is characterised by a value of H = 0.76, while H = 0.82 for cluster 2. Cluster 1 is also characterised by a <DSC> median value of 0.48 kHz lower than for cluster 2 with <DSC> median values of about 0.52 kHz (see Table 6). In both clusters, this indicator reveals that the frequency content is shifted towards the low frequency range.

The three-cluster solution highlights Site 18 which contains distinctive eco-acoustic indices from the previous cluster 2. This can be clearly observed by looking at Figure 10 central column (see indices ADI, AEI, H, and DSC). What emerges is the surge of DSC index passing from 0.52 kHz for cluster 2 to 0.98 KHz for Site 18 (cluster 3) (see Table 7). The four-cluster solution presents a split of cluster 1. In this case, the eco-acoustics’ indices ADI and H seems to benefit from this further subdivision as they appear much separated, thus revealing the presence of less frequency abundance for cluster 2 (2 out of 4) than cluster 1 (see Figure 10 right column and Table 8).

As it is apparent from Figure 11a, <ADI>, which drives (together the correlated AEI and H indices) the clustering process, reflects a similar distribution to that obtained from a two-cluster solution. In particular, sites close to the main traffic noise source are poor in components at mid-high frequency (lower ADI values). Site 18 is highlighted by the three-cluster solution. Such information is actually carried by the DSC index, as illustrated in Figure 11b.

**Figure 14:**
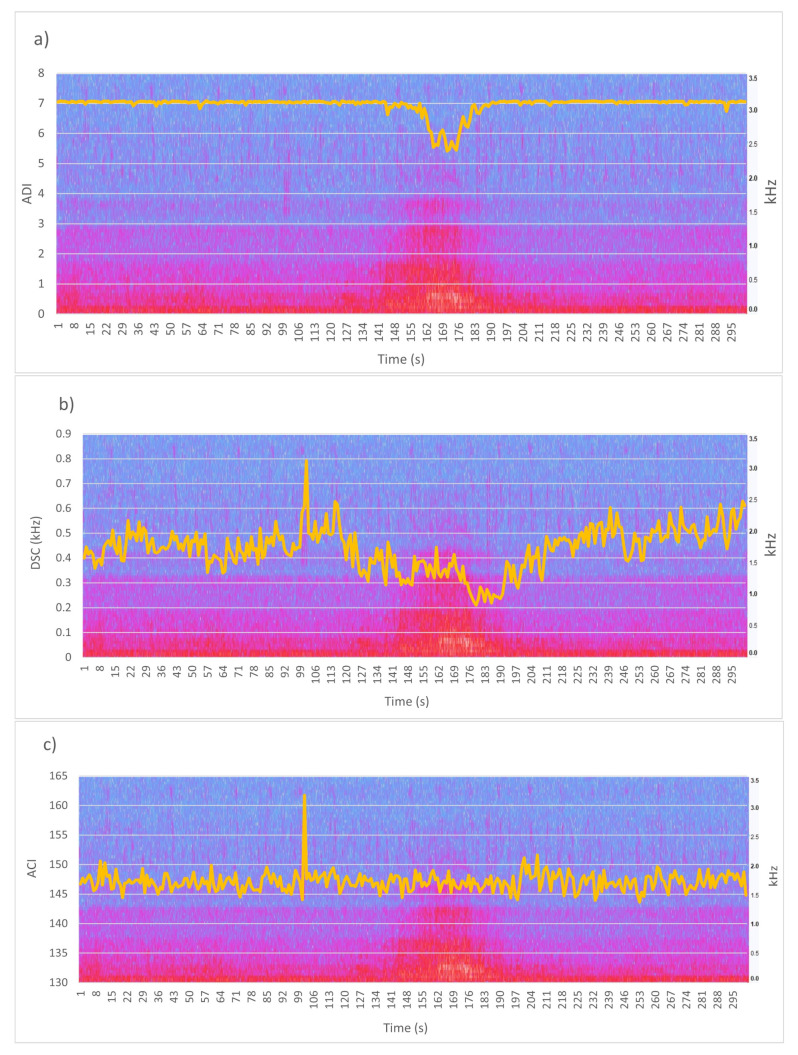
Spectrogram of a take-off overlapped with the ADI (**a**), DSC (**b**), and ACI (**c**) indices. Time scale 0–5 min.

We also analysed the potential effect of the nearby airport traffic. During the measurement period, 20 airplanes taking off were counted. Their effects on the eco-acoustic indices were evaluated with reference to Site 17 which sits at a distance of approximately 700 m from the runway. Figure 14 shows the spectrogram of a take-off overlapped with the ADI, DSC and ACI indices. As it is apparent, ADI and DSC are affected by the transient noise whereas ACI seems not to be sensitive to it.

This result is confirmed by calculating the indices’ value over a one-minute interval before, during (at 01:41), and after take-off, as shown in Table 9.

**Figure 15:**
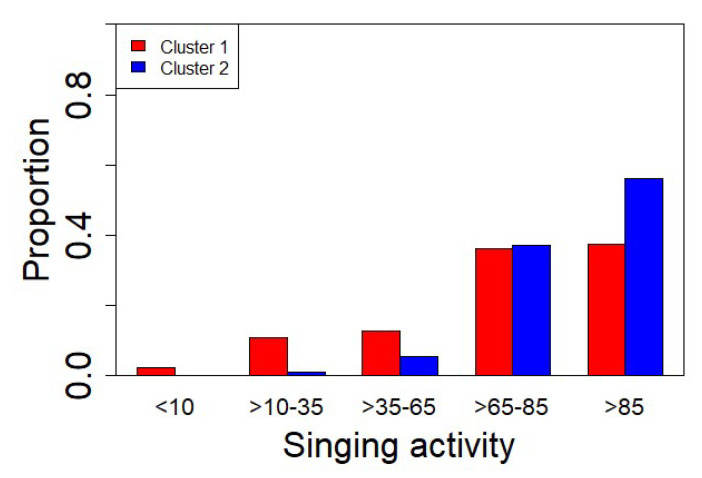
Proportion of recordings falling in each cluster and in each interval of singing activity expressed as a percentage with respect to the recording duration (1 min).

In order to provide a more solid interpretation of the results emerging from the cluster analysis, we checked for all the aural characteristics highlighted by an expert operator. In Figure 15, we illustrate the singing activity quantified as the percentage of singing presence in the audio recording time. In cluster 1, we have the presence of a significant proportion (26%) of recordings with low/moderate bird activity (<65%), an interval of activity between 65% and 85%, with a proportion of 36% equally distributed in the two clusters and a prevalence of recordings with singing activity above 85% of 56% for cluster 2 against 37% for cluster 1.

**Figure 16:**
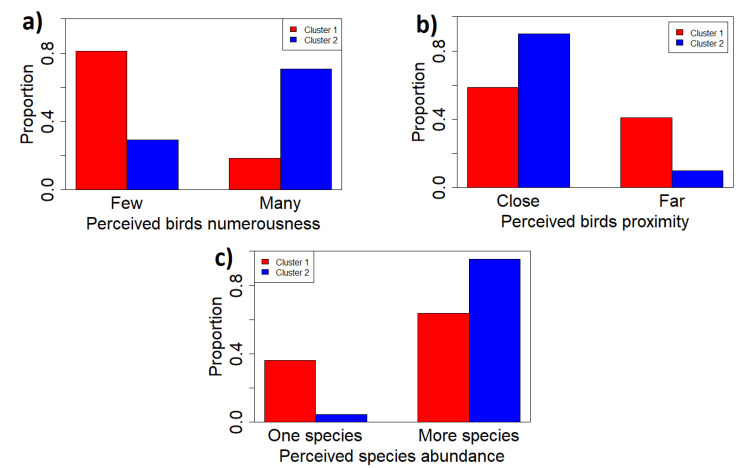
(**a**) Perceived singing abundance in the two clusters; (**b**) perceived singing distance in the two clusters; and (**c**) perceived abundance of species in the two clusters.

The perceived singing abundance in the two clusters, shown in Figure 16a, confirms the previous results: there was singing activity in the majority of recordings in cluster 1 (∼80%) and many bird were singing for the majority of cluster 2 (∼70%). As for the perceived singing distance, both clusters’ composition is made of audio recordings containing close and distant singing activity but with very different proportions. Cluster 1 contains a proportion of close perceived singing activity of ∼60% and ∼40% of distant perceived singing activity, whereas in cluster 2, we find ∼90% and ∼10%, respectively, (see Figure 16b). The presence of a richer singing population for sites belonging to cluster 2 is evidenced in Figure 16c. In particular, for cluster 2 the presence of many species reaches the value of ∼95% against ∼60% for cluster 1.

**Figure 17:**
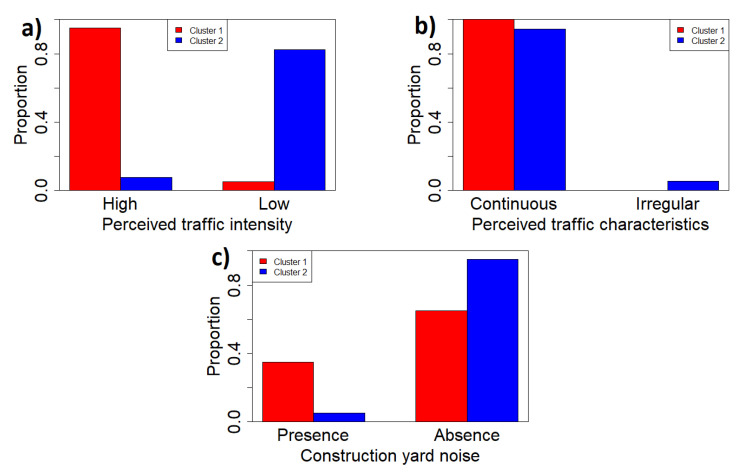
(**a**) Perceived traffic intensity in the two clusters; (**b**) perceived traffic characteristics in the two clusters; and (**c**) perceived construction yard noise in the two clusters.

Figure 17a,b report the proportion of audio recordings in the two clusters versus the presence of traffic perceived in terms of two categories: intensity and dynamic characteristics. The results seem to indicate that cluster 1 presents the majority of audio recordings characterised by high perceived intensity (which can be also addressed as a close perceived traffic distance), whereas for cluster 2, the perception is of a low intensity traffic noise (distant traffic noise). For both clusters, the traffic noise is, however, perceived as continuous. Figure 17c illustrates the presence of acoustic sources from a construction site characterised by moving-vehicle-alarm beeps and impact noises. In this case, cluster 2 shows the majority of recordings without such a kind of disturbance (95%) against cluster 1 (65%).

In order to determine the potential influence of the vegetation coverage as a biophonic activity driver, in Table 10, we report the mean NDVI values, <NDVI>, computed for the two-, three-, and four-cluster solution illustrated in Figure 13. As is apparent, <NDVI> values are almost similar in all clusters. This result may suggest that, for the considered situation, the vegetation coverage is not the main cause of the observed results, in contrast to what is observed in another parcel of the park as described in [27].

As a final remark, the analysis on the remaining time slots, namely from 26 May 2015 to 29 May 2015, confirms the results presented above in terms of sites membership in the different clusters. This fact underlines the robustness of the analysis and the stationarity of the sound environment during the analysed period.

## 5. Conclusions

Urban parks are often exposed to road traffic noise produced by the surrounding roads and other anthropogenic sounds due to the presence of people and noises from construction sites. Such sounds alter the natural soundscape, reducing the overall sound quality of a park, thus interfering with the presence of biophonic activity either due to the partial overlapping of frequencies or high background noise levels. For this reason, assessing the health of urban ecosystems and extending the concept of the soundscape, previously limited to residential urban areas, also to urban green areas using an eco-acoustic analysis approach, would help monitor biodiversity estimation and obtain useful information for conservation and development planning.

The availability of very low-cost sound recorders allowed drawing a real picture of the sound environment over an extended area with a cost-effective solution. In particular, we focused on studying the sound characteristics collected at 16 sites at the Parco Nord located in the city area of Milan, Italy, using very low-cost sensors distributed over an area of nearly 20 hectares. Statistics, PCA and cluster analysis, applied to five eco-acoustic indices, allowed identifying areas (clusters) with different sound characteristics.

What drives the cluster formation is essentially Dimension 1 of the PCA with 54.1% of explained variance and represented by the correlated indices ADI, AEI, and H. The spatial distribution of ADI (see Figure 11a) retraces the results of the cluster analysis: a spectrum with less frequency occupation close to the highway and dominated by traffic noise sources which becomes richer in frequencies as we move along into the park. Dimension 2 (DSC in Figure 11b) highlights site 18 as characterised by a higher spectral centroid. These characteristics are picked up when considering the three-cluster solution. Finally, Dimension 3 emphasises how frequency modulations are mostly concentrated in the inner part of the studied park area.

In general, two main areas emerge from the statistical analysis. The first (cluster 1) is characterised by less frequency modulation, frequency richness, and with an overall lower spectral centroid, typical of areas with less biophonic activity and higher anthropophonic disturbances (traffic noise and construction site). Cluster 2 presents higher index values as a result of a greater distance from non-natural sources and a more lively biophonic presence. This result is confirmed by the aural survey revealing a more intense and richer singing activity in cluster 2 and with a traffic noise perceived as continuous and less intense. The analysis performed on the other recording time-slots (26–29 May 2015) confirms the results, as illustrated throughout the paper (data of 25 May 2015), suggesting both the robustness of the analysis and the stationarity of the sound environment during the short period under analysis.

Additionally, the NDVI index does not seem to provide evidence that the vegetation coverage is the main cause of the observed results, as it does not change significantly within the areas associated with each cluster. Owing to the limited extension of the recorded tracks (3.5 h), this work represents a feasibility study, showing the potentiality of the adopted method to describe the acoustic complexity of an urban park and highlighting areas with different sound components. The inherent sound changes involved in environmental dynamics could take advantage of this approach, which can be used to evaluate ecosystem health trends over long periods.

In general, mapping environmental sound over large areas by means of eco-acoustic indices could be applied as an innovative diagnostic tool able to account for the effect of climate change in different biomes. The sensitivity of the sonic performances of animals even under the modest modification of the physical parameters of the environment (e.g., temperature, humidity and pH for the aquatic medium) allows to investigate with great efficiency the effects of human intrusion and climate change. The fine scale of resolution of the eco-acoustics approach can be easily integrated with the new generation of satellite and terrestrial remote sensing techniques (e.g., LIDAR). The initial effects of global change on physiology and the behaviour of organisms can be evaluated, saving important time otherwise requested to detect the effects on the environment at a scale of decades.

The information gained by eco-acoustics surveys can be used as anticipatory tools to suggest immediate actions and producing precious indications to stakeholders and policymakers before irreversible environmental changes occur, saving time and economic resources. The analysis of the collected recordings is oriented to discover and describe eco-acoustic events of relevance for better understanding the dynamics activated by climate change. The latter is an important point to encourage the world-wide diffusion of this approach that requires a relatively modest investment in ground-based technology. As future development of this work, we are planning to extend this approach to two different habitats, namely Parco del Ticino, a natural park of north Italy, and along the marine coastline of Liguria sea with the goal of validating the methodologies to use eco-acoustic information on the large scale to optimise the investigation and monitor the evolution of the two sound environments.

## Figures and Tables

**Table 1:** Sound sources considered in the aural survey.

Category	Sound Source
Biophony	Bird, dog, frog
Technophony	Road traffic, construction site, sirens, airplanes
Geophony	Rain, wind
Antropophony	Voice, step

**Table 2:** Perceived indicators and corresponding labelling adopted for biophonic and technophonic sources.

Perceived Indicators	Labelling				
**Abundance**	**No Birds**		**Few Birds**		**Many Birds**
Activity	[0–10]%	(10–35]%	(35–65]%	(65–85]%	(85–100]%
Traffic Type	Continuous			Irregular	

**Table 3:** Calculated indices with reference to Figure 7.

	ACI	ADI	AEI	H	DSC
Site 2	164.22	5.88	0.76	0.73	0.53
Site 17	164.78	6.94	0.31	0.84	0.71

**Table 4:** Percentage contribution of each eco-acoustic index to the first three principal components (dimensions).

Contribution (%)	ACI	ADI	AEI	DSC	H
Dimension 1	5.2	29.8	30.0	7.5	27.4
Dimension 2	16.6	9.8	7.6	57.8	8.3
Dimension 3	41.5	3.6	5.9	34.5	14.6

**Table 5:** Wilcoxon–Mann–Whitney test applied to the two-cluster solution. *p*-value and test outcome at 95% significance level.

Index	*p*-Value	Test Outcome
ACI	0.114	Failed to reject
ADI	0.000175	Rejected
AEI	0.000175	Rejected
H	0.000175	Rejected
DSC	0.21	Failed to reject

**Table 6:** Median values of the eco-acoustic indices in clusters 1 and 2 obtained with k-means algorithm.

	ACI	ADI	AEI	H	DSC (kHz)
Cluster 1	146.97	6.64	0.47	0.76	0.48
Cluster 2	147.09	6.96	0.23	0.82	0.52

**Table 7:** Median values of the eco-acoustic indices in clusters 1, 2, and 3 obtained with DIANA and k-means algorithms.

	ACI	ADI	AEI	H	DSC (kHz)
Cluster 1	146.97	6.64	0.47	0.76	0.48
Cluster 2	147.06	6.96	0.23	0.82	0.52
Cluster 3	147.57	7.06	0.09	0.58	0.98

**Table 8:** Median values of the eco-acoustic indices in clusters 1, 2, and 3 obtained with DIANA and k-means algorithms.

	ACI	ADI	AEI	H	DSC (kHz)
Cluster 1	146.99	6.76	0.40	0.76	0.48
Cluster 2	146.09	6.37	0.58	0.73	0.48
Cluster 3	147.06	6.96	0.23	0.82	0.52
Cluster 4	147.57	7.06	0.09	0.87	0.98

**Table 9:** Indices value calculated over a one-minute interval before, during (at 01:41), and after take-off.

Time (hh:mm)	ADI	DSC (kHz)	ACI
01:40	7.04	0.47	147.23
01:41	6.63	0.35	146.96
01:42	7.01	0.40	147.15

**Table 10:** <NDVI> values computed for the areas corresponding to two-, three-, and four-cluster solutions illustrated in Figure 13.

Number of Clusters	Cluster 1	Cluster 2	Cluster 3	Cluster 4
2	0.774	0.771	-	-
3	0.774	0.771	0.777	-
4	0.778	0.767	0.771	0.777

## Data Availability

Data available upon request.

## References

[1] Parker D.E. (2010). Urban heat island effects on estimates of observed climate change. Wiley Interdiscip. Rev. Clim. Chang..

[2] Ghaffarianhoseini A., Dahlan N.D., Berardi U., Makaremi N., Ghaffarianhoseini M. (2013). Sustainable energy performances of green buildings: A review of current theories, implementations and challenges. Renew. Sustain. Energy Rev..

[3] Gunnarsson B., Knez I., Hedblom M., Sang Å.O. (2017). Effects of biodiversity and environment-related attitude on perception of urban green space. Urban Ecosyst..

[4] Irvine K.N., Devine-Wright P., Payne S.R., Fuller R.A., Painter B., Gaston K.J. (2009). Green space, soundscape and urban sustainability: An interdisciplinary, empirical study. Local Environ..

[5] Allen C., Forys E. (2005). The impacts of sprawl on biodiversity: The ant fauna of the lower Florida Keys. Ecol. Soc..

[6] Barber J.R., Crooks K.R., Fristrup K.M. (2010). The costs of chronic noise exposure for terrestrial organisms. Trends Ecol. Evol..

[7] Lawson G.M., Qun F., Brearley J. (2011). Networks cities and ecological habitats. Networks Cities.

[8] Staaterman E., Rice A.N., Mann D.A., Paris C.B. (2013). Soundscapes from a Tropical Eastern Pacific reef and a Caribbean Sea reef. Coral Reefs.

[9] Sueur J., Farina A. (2015). Ecoacoustics: The ecological investigation and interpretation of environmental sound. Biosemiotics.

[10] Farina A. (2013). Soundscape Ecology—Principles, Patterns Methods and Applications.

[11] Krause B. (1987). Bioacoustics, habitat ambience in ecological balance. Whole Earth Rev..

[12] Pijanowski B.C., Farina A., Gage S.H., Dumyahn S.L., Krause B.L. (2011). What is soundscape ecology? An introduction and overview of an emerging new science. Landsc. Ecol..

[13] Sueur J., Farina A., Gasc A., Pieretti N., Pavoine S. (2014). Acoustic indices for biodiversity assessment and landscape investigation. Acta Acust. United Acust..

[14] Kasten E.P., Gage S.H., Fox J., Joo W. (2012). The remote environmental assessment laboratory’s acoustic library: An archive for studying soundscape ecology. Ecol. Inform..

[15] Eldridge A., Guyot P., Moscoso P., Johnston A., Eyre-Walker Y., Peck M. (2018). Sounding out ecoacoustic metrics: Avian species richness is predicted by acoustic indices in temperate but not tropical habitats. Ecol. Indic..

[16] Boelman N.T., Asner G.P., Hart P.J., Martin R.E. (2007). Multitrophic invasion resistance in hawaii: Bioacoustics, field surveys, and airborne remote sensing. Ecol. Appl..

[17] Harris S.A., Shears N.T., Radford C.A. (2016). Ecoacoustic indices as proxies for biodiversity on temperate reefs. Methods Ecol. Evol..

[18] Bertucci F., Parmentier E., Berten L., Brooker R.M., Lecchini D. (2015). Temporal and spatial comparisons of underwater sound signatures of different reef habitats in Moorea Island, French Polynesia. PLoS ONE.

[19] Sueur J., Pavoine S., Hamerlynck O., Duvail S. (2008). Rapid acoustic survey for biodiversity appraisal. PLoS ONE.

[20] Krause B., Farina A. (2016). Using eco-acoustic methods to survey the impacts of climate change on biodiversity. Biol. Conserv..

[21] Manvell D., Ballarin Marcos L., Stapelfeldt H., Sanz R. SADMAM-Combining Measurements and Calculations to Map Noise in Madrid. Proceedings of the Inter-Noise 2004.

[22] Benocci R., Bellucci P., Peruzzi L., Bisceglie A., Angelini F., Confalonieri C., Zambon G. (2019). Dynamic noise mapping in the suburban area of Rome (Italy). Environments.

[23] De Coensel B., Sun K., Wei W., Van Renterghem T., Sineau M., Ribeiro C., Can A., Aumond P., Lavandier C., Botteldooren D. Dynamic noise mapping based on fixed and mobile sound measurements. Proceedings of the 10th European Congress and Exposition on Noise Control Engineering (Euronoise 2015).

[24] Wei W., Van Renterghem T., De Coensel B., Botteldooren D. (2016). Dynamic noise mapping: A map-based interpolation between noise measurements with high temporal resolution. Appl. Acoust..

[25] Zambon G., Benocci R., Angelini F., Brambilla G., Gallo V. Statistics-based functional classification of roads in the urban area of Milan. Proceedings of the 7th Forum Acusticum.

[26] Benocci R., Roman H.E., Bisceglie A., Angelini F., Brambilla G., Zambon G. (2021). Eco-acoustic assessment of an urban park by statistical analysis. Sustainability.

[27] Benocci R., Roman H.E., Bisceglie A., Angelini F., Brambilla G., Zambon G. (2022). Auto-correlations and long time memory of environment sound: The case of an Urban Park in the city of Milan (Italy). Ecol. Indic..

[28] Benocci R., Brambilla G., Bisceglie A., Zambon G. (2020). Eco-Acoustic Indices to Evaluate Soundscape Degradation Due to Human Intrusion. Sustainability.

[29] Landsat-8 Mission. https://landsat.gsfc.nasa.gov/satellites/landsat-8/.

[30] Carlson T., Ripley D.A. (1997). On the Relation between NDVI, Fractional Vegetation Cover, and Leaf Area Index. Remote Sens. Environ..

[31] QGIS 3.16.0 [Computer Software]. QGIS Geographic Information System. QGIS Association. https://download.qgis.org/downloads/.

[32] Vegetation Index. https://www.agricolus.com/indici-vegetazione-ndvi-ndmi-istruzioni-luso/.

[33] R Core Team (2018). R: A Language and Environment for Statistical Computing.

[34] Seewave: Sound Analysis and Synthesis. https://cran.r-project.org/web/packages/seewave/index.html.

[35] Soundecology: Soundscape Ecology. https://cran.r-project.org/web/packages/soundecology/index.html.

[36] Pieretti N., Farina A., Morri D. (2011). A new methodology to infer the singing activity of an avian community: The Acoustic Complexity Index (ACI). Ecol. Indic..

[37] Grey J.M., Gordon J.W. (1978). Perceptual effects of spectral modifications on musical timbres. J. Acoust. Soc. Am..

[38] Yang W., Kang J. (2005). Soundscape and sound preferences in urban squares: A case study in Sheffield. J. Urban Des..

[39] Hervé Abdi H., Williams L.J. (2010). Principal component analysis. Comput. Stat..

[40] Jolliffe I.T., Cadima J. (2016). Principal component analysis: A review and recent developments. Philos. Trans. R. Soc. A Math. Phys. Eng. Sci..

[41] Cluster Analysis. https://en.wikipedia.org/wiki/Cluster_analysis.

[42] Ward J.H. (1963). Hierarchical grouping to optimize an objective function. J. Am. Stat. Assoc..

[43] Hartigan J.A., Wong M.A. (1979). A k-means clustering algorithm. Appl. Stat..

[44] Kaufman L., Rousseeuw P. (1990). Finding Groups in Data.

[45] Herrero J., Valencia A., Dopazo J. (2001). A hierarchical unsupervised growing neural network for clustering gene expression patterns. Bioinformatics.

[46] Package ‘clValid’. https://cran.r-project.org/web/packages/clValid/clValid.pdf.

[47] Brock G., Pihur V., Datta S., Datta S. (2008). clValid: An R package for cluster validation. J. Stat. Softw..

[48] Handl J., Knowles J., Kell D.B. (2005). Computational cluster validation in post-genomic data analysis. Bioinformatics.

[49] Dunn J.C. (1974). Well separated clusters and fuzzy partitions. J. Cybern..

[50] Rousseeuw P.J. (1987). Silhouettes: A graphical aid to the interpretation and validation of cluster analysis. J. Comput. Appl. Math..

[51] Datta S. (2003). Comparisons and validation of statistical clustering techniques for microarray gene expression data. Bioinformatics.

[52] Yeung K.Y., Haynor D.R., Ruzzo W.L. (2001). Validating clustering for gene expression data. Bioinformatics.

[53] Pihur V., Datta S., Datta S. (2007). Weighted rank aggregation of cluster validation measures: A Monte Carlo cross-entropy approach. Bioinformatics.

[54] Borg I., Groenen P. (2005). R: Modern Multidimensional Scaling: Theory and Applications.

[55] Conover W.J. (1999). Practical Nonparametric Statistics.

[56] Kruskal W. (1952). Use of ranks in one-criterion variance analysis. J. Am. Stat. Assoc..

[57] Siegel C. (1988). Nonparametric Statistics for the Behavioral Sciences.

[58] Shapiro S.S., Wilk M.B. (1965). An analysis of variance test for normality (complete samples). Biometrika.

